# Cellular and molecular defects in a patient with Hermansky-Pudlak syndrome type 5

**DOI:** 10.1371/journal.pone.0173682

**Published:** 2017-03-15

**Authors:** Joshi Stephen, Tadafumi Yokoyama, Nathanial J. Tolman, Kevin J. O’Brien, Elena-Raluca Nicoli, Brian P. Brooks, Laryssa Huryn, Steven A. Titus, David R. Adams, Dong Chen, William A. Gahl, Bernadette R. Gochuico, May Christine V. Malicdan

**Affiliations:** 1 Medical Genetics Branch, National Human Genome Research Institute, National Institutes of Health, Bethesda, Maryland, United States of America; 2 NIH Undiagnosed Diseases Program, Common Fund, Office of the Director, National Institutes of Health, Bethesda, Maryland, United States of America; 3 Ophthalmic Genetics & Visual Function Branch, National Eye Institute, National Institutes of Health, Bethesda, Maryland, United States of America; 4 Division of Pre-clinical Innovation, National Center for Advancing Translational Sciences, National Institutes of Health, Rockville, Maryland, United States of America; 5 Office of the Clinical Director, National Human Genome Research Institute, National Institutes of Health, Bethesda, Maryland, United States of America; 6 Division of Hematopathology, Mayo Clinic, Rochester, Minnesota, United States of America; Purdue University, UNITED STATES

## Abstract

Hermansky-Pudlak syndrome (HPS) is a heterogeneous group of genetic disorders typically manifesting with tyrosinase-positive oculocutaneous albinism, bleeding diathesis, and pulmonary fibrosis, in some subtypes. Most HPS subtypes are associated with defects in Biogenesis of Lysosome-related Organelle Complexes (BLOCs), which are groups of proteins that function together in the formation and/or trafficking of lysosomal-related endosomal compartments. BLOC-2, for example, consists of the proteins HPS3, HPS5, and HPS6. Here we present an HPS patient with defective BLOC-2 due to a novel intronic mutation in *HPS5* that activates a cryptic acceptor splice site. This mutation leads to the insertion of nine nucleotides in-frame and results in a reduced amount of HPS5 at the transcript and protein level. In studies using skin fibroblasts derived from the proband and two other individuals with HPS-5, we found a perinuclear distribution of acidified organelles in patient cells compared to controls. Our results suggest the role of HPS5 in the endo-lysosomal dynamics of skin fibroblasts.

## Introduction

Hermansky-Pudlak syndrome (HPS) is a group of related autosomal recessive disorders due to mutations in genes involved in intracellular membrane and protein trafficking. HPS was first reported in 1959 by Hermansky and Pudlak, who described two patients with oculo-cutaneous albinism and prolonged bleeding [1]. Currently, OMIM (On line Mendelian Inheritance of Man) describes 10 genetic subtypes of HPS: type 1 (due to mutations in *HPS1*), type 2 (*AP3B1*), type 3 (*HPS3*), type 4 (*HPS4*), type 5 (*HPS5*), type 6 (*HPS6*), type 7 (*DTNBP1*), type 8 (*BLOC1S3*), type 9 (*BLOC1S6*), and type 10 (*AP3D1*) [2]. Products of 8 of the 10 HPS genes operate in distinct protein complexes called the Biogenesis of Lysosome-related Organelle Complexes (BLOCs) that function together in the formation and/or trafficking of lysosome-related endosomal compartments [3]; the products of *AP3B1* and *AP3D1* are subunits of adaptor protein complex-3 (AP-3), which plays a role in enriching cargo proteins in vesicles for transport through the intracellular endosomal/lysosomal pathway [4].

The trans Golgi network is the first-round sorting center of newly synthesized molecules destined for the lysosome, melanosome, and other lysosome-related organelles (LRO) [5]. Golgi-derived proteins destined for LROs and late endosomes/lysosomes, and proteins of the recycling endosomal pathway are also sorted in early endosomes and their associated tubules [3]. HPS protein complexes (eg: BLOCs) contribute to the maturation of organelles by regulating the delivery of molecules to LROs. BLOC-1 is a multimeric complex including HPS7, HPS8 and HPS9 [6]; BLOC-2 comprises HPS3, HPS5 and HPS6 [7], and HPS1 and HPS4 form BLOC-3 [8]. Human HPS subtypes with mutations in the same BLOC manifest similar phenotypes, and the severity of the phenotype varies according to the type of BLOC defect [6]. For example, pulmonary fibrosis is associated with HPS types 1 and 4 (BLOC-3), while neutropenia, absence of lytic granules in lymphocytes, immunodeficiency, and interstitial fibrosis are characteristics of HPS type 2 [3].

In this study, we report a patient with a milder form of HPS resulting from defective BLOC-2 due to a novel intronic mutation in the *HPS5* gene. We also describe the consequences of this mutation at the cellular and molecular levels.

## Materials and methods

Written informed consent was provided for clinical protocols 95-HG-0195 (“Clinical and Basic Investigations into Hermansky-Pudlak Syndrome”) and 04-HG-0211 (“Procurement and Analysis of Specimens from Individuals with Pulmonary Fibrosis”), which were approved by the National Human Genome Research Institute (NHGRI) Institutional Review Board. Clinical evaluations, including high-resolution computed tomography scans of the chest and bronchoscopy with lavage, were performed at the National Institutes of Health (NIH) Clinical Center as previously described [9].

### Targeted panel sequencing

Exome sequencing was performed at the Casey Eye Institute Molecular Diagnostic Laboratory using Pigmentation SmartPanel (v3; gene list is available upon request). Direct testing for mutations in the genes of the Pigmentation Smart Panel was performed by PCR amplification and Next Generation Sequencing. PCR primer sets were printed on the SmartPanel chips. Each primer set is duplicated on the chips in order to avoid random PCR failure. Any low coverage region (<100X) was covered afterwards by PCR and Sanger sequencing. Identified mutations and novel variants were confirmed by Sanger sequencing. For dideoxy sequencing, specific primers were designed (primer sequences available upon request) to amplify the desired regions. Direct sequencing of the PCR amplification products was performed using BigDye 3.1 Terminator chemistry (Applied Biosystems, Austin, Texas, USA) and separated on an ABI 3130xl genetic analyzer (Applied Biosystems, Foster city, CA, USA). Data were evaluated using Sequencher v5.0 software (Gene Codes Corporation, Ann Arbor, MI).

### Cell culture

Fibroblasts were cultured from forearm skin biopsies and grown in high-glucose (4.5 g/l) DMEM supplemented with 15% FBS, 2 mM L-glutamine, nonessential amino acid solution, and penicillin-streptomycin. Normal adult human dermal fibroblasts (ATCC® PCS-201-012™) served as controls.

### Reverse transcriptase PCR, cDNA analysis and qPCR

Total RNA was isolated from the skin-derived fibroblast culture using the RNeasy Mini Kit (Qiagen, Valencia, CA) according to the manufacturer’s protocol. RNA concentration and purity were assessed and first strand cDNA was synthesized using a high capacity RNA-to-cDNA kit (Applied Biosystems). Reverse transcriptase reaction products were amplified using specific primers (forward 5’-AAGAAGGCTGGAAGCACAGG-3’ and reverse 5’-TCCCACATCCTAGAGCCTGG-3’) and loaded into 2% agarose gel. Sanger sequencing of the excised bands was performed as mentioned above. Quantitative real-time PCR (qPCR) was performed using primers specific for the three different isoforms of *HPS5*. cDNA derived from both patient and control was amplified using Power SYBR Green PCR master mix (Applied Biosystems, Woolston, Warrington, UK) and the real-time amplification was monitored using Bio-Rad qPCR machine (CFX96 Touch Real Time PCR detection system, Philadelphia, PA, USA) with standard qPCR parameters. Results were normalized with expression of the housekeeping gene *POLR2A* and analyzed using the comparative C_T_ method [10]

### Western blotting

Fibroblasts were grown to confluency in T75 flasks, trypsinized, and pelleted. Total protein from cell pellets was obtained by lysing in RIPA buffer (Sigma-Aldrich) containing protease inhibitor cocktail (Sigma-Aldrich). The total amount of protein in each sample was determined using the BCA assay kit (Pierce, Rockford, IL). 20μg of the proteins were electrophoresed on a 4–12% Bis-Tris or 3–8% Tris-Acetate gel (Life Technologies) with MOPS, MES, or Tris-Acetate buffer (Life Technologies) and blotted onto a PVDF or nitrocellulose membrane (Invitrogen, Carlsbad, CA, USA). Membranes were blocked with Li-Cor blocking buffer (Li-Cor Biosciences) for one hour, and then incubated overnight at 4°C with the following antibodies specific for each protein: rabbit polyclonal anti-HPS5 Ab (Proteintech), rabbit polyclonal anti-HPS6 Ab (Origene), rabbit polyclonal anti-HPS3 Ab (Proteintech), rabbit polyclonal anti-HPS2 Ab (Proteintech), rabbit polyclonal anti-HPS4 Ab (Proteintech), mouse monoclonal anti-HPS7 Ab (Santa Cruz Biotech), rabbit polyclonal anti-Rab11 (Abcam), mouse monoclonal anti-β-actin Ab (CST), rabbit polyclonal anti-β-actin Ab (Abcam), and mouse monoclonal anti-β-actin Ab (Sigma). After 3 washes in PBS with 0.1% Tween-20, membranes were incubated with the appropriate secondary antibodies (Li-Cor Biosciences, Lincoln, NE). The target bands were scanned and visualized using Odyssey^®^ imaging system (Li-COR).

### Immunofluorescence microscopy

Fibroblasts were cultured in chamber slides for 72 hrs after passage and washed with PBS twice, fixed with 4% PFA at room temperature for 15 min, and permeabilized using 0.1% saponin. Slides were blocked with 5% donkey serum and 1% BSA for 30 min, incubated in primary Abs overnight at 4°C followed by incubation in appropriate secondary Abs, mounted in Vectashield solution containing DAPI (Vector Laboratories). Antibodies used for this method were: rabbit polyclonal anti-EEA1 Ab (Abcam), rabbit polyclonal anti-Rab11 Ab (Abcam), rabbit monoclonal anti-Rab7 AB (Cell Signaling), mouse monoclonal anti-CD63/LAMP3 Ab (H5C6; Developmental Studies, Hybridoma Bank), and Alexa fluor secondary donkey anti-rabbit/mouse Abs (Invitrogen). Cells were imaged with a Zeiss 510 META confocal laser-scanning microscope. For quantification of CD63 staining, 6000 cells/well were seeded in 96-well black/clear bottom plates and stained with anti-CD63/LAMP3 Ab, followed by donkey anti-mouse Alexa fluor secondary antibody. Cells were then imaged on the InCell 2200 Analyzer and quantified as described elsewhere [11].

### LysoTracker assay

LysoTracker red dye stains acidic cellular environment and visualizes acidic late endosomes and lysosomes. 6000 cells/well were seeded in 96-well black/clear bottom plates and stained using LysoTracker Red dye (L-7538, Life Technologies), and imaged as above using the InCell 2200 Analyzer.

### Statistical analysis

Data shown are ± standard deviation or standard error of the mean as noted. Student’s t-test was performed to analyze significance of difference between means (GraphPad Prism, San Diego, CA).

## Results

### Clinical report

The patient is a 27-year-old Turkish man evaluated at the NIH Clinical Center for oculocutaneous albinism. He had mild hypopigmentation, nystagmus, and impaired visual acuity, but could read with corrective lenses. He noted easy bruising and prolonged bleeding following extractions of his teeth, and had a history of recurrent epistaxis; laser surgery decreased the frequency of his epistaxis. He never received medications to treat excessive bleeding or transfusions of blood products. He had no history of unusual infections, dyspnea, cough, abdominal pain, hematochezia, or abnormal bowel movements. His parents were first cousins but, although his sister, paternal uncle, and several first cousins had albinism, there was no known family history of HPS.

**Fig 1:**
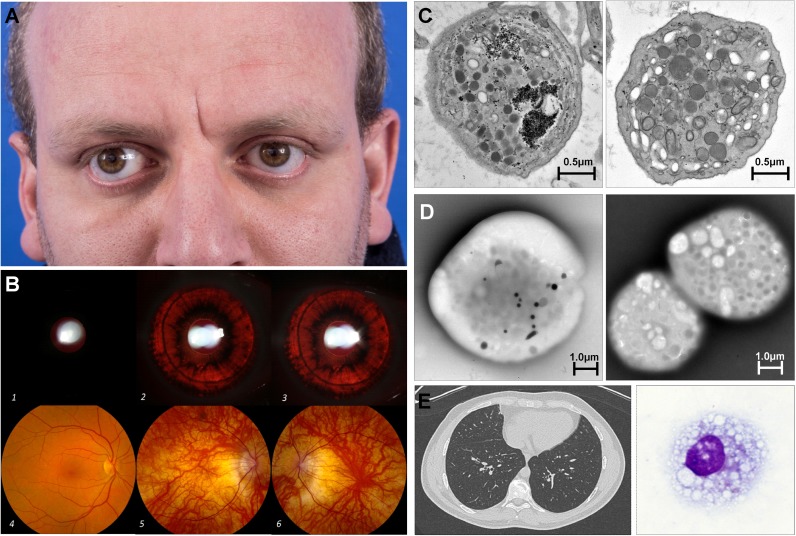
Clinical characteristics of the patient. **(A)** Photograph showing skin and hair color of the patient (**B)** Iris transillumination: 1, normal right iris; patient’s iris with transillumination defects (2, right eye; 3, left eye); Fundus: 4, normal fundus right eye; patient’s poorly pigmented fundi with peripapillary atrophy and foveal hypoplasia (5, right eye; 6, left eye) **(C)** Electron microscopy (EM) findings of the thin sections of platelets imaged by transmission EM, showing platelet from control (left panel) and patient (right panel) **(D)** whole mount EM of platelets showing lack of delta-granules in patient (right panel) compared to control (left panel) **(E)** Computed tomography scan of the chest shows no evidence of pulmonary fibrosis (left panel); right panel showing foamy appearance of alveolar macrophages in bronchoalveolar lavage fluid.

On physical examination, the patient had fair skin with some degree of sun tanning. His hair color was a medium to dark brown (Fig 1A). He had nystagmus, a V-pattern exotropia, and best corrected visual acuity of 20/125 bilaterally. Ophthalmologic examination revealed iris trans-illumination and a fundus with minimal retinal pigmentation compared to normal (Fig 1B). He also had peripapillary atrophy in the setting of high myopia. Optical coherence tomography demonstrated a poorly developed fovea consistent with foveal hypoplasia and visual evoked potential testing revealed crossing changes typically seen in patients with albinism. Pulmonary, cardiac, abdominal and neurological physical examination findings were normal.

Laboratory testing revealed normal chemistries, serum glucose, and liver function tests. Blood urea nitrogen and serum creatinine were normal, and 24-hour creatinine clearance was 156 ml/minute [normal range 90–125 ml/minute]. Complete blood count showed normal white blood cell count and differential, hemoglobin, and hematocrit; platelet count was 200 K/ul [normal range 161–347 K/ul]. Prothrombin time and activated partial thromboplastin time were normal. Electrocardiogram demonstrated sinus rhythm, and echocardiogram showed normal ventricular wall motion and no evidence of pulmonary hypertension.

Given the history of oculocutaneous albinism, nystagmus and tendency for bleeding, the patient was evaluated for HPS. Thin section electron microscopy images showed that the patient’s platelets have no gross abnormalities in overall granule content (Fig 1C). Whole mount transmission electron microscopy of platelets demonstrated absent dense bodies or delta granules (Fig 1D), consistent with the diagnosis of HPS. Pulmonary function testing revealed a forced vital capacity 109% of predicted and diffusion capacity 73% of predicted. Oxygen saturations remained 99% throughout a six-minute walk test. High-resolution computed tomography scan of the chest showed no evidence of interstitial lung disease or ground glass opacification (Fig 1E, left panel). Bronchoalveolar cell differential count showed 93% alveolar macrophages, 5% lymphocytes, and 2% polymorphonuclear leukocytes. Imaging of stained bronchoalveolar cells revealed 16.5% (46/300 cells) of total to be foamy alveolar macrophages (Fig 1E, right panel). We analyzed the morphology of alveolar macrophages from an adult patient with HPS-1, who was closely matched for age and gender with our patient. We found that 18 (6.4%) of 307 alveolar macrophages were foamy in this patient with HPS-1, who did not have interstitial lung disease but is at high risk for future development of pulmonary fibrosis.

### Mutation identification

**Fig 2:**
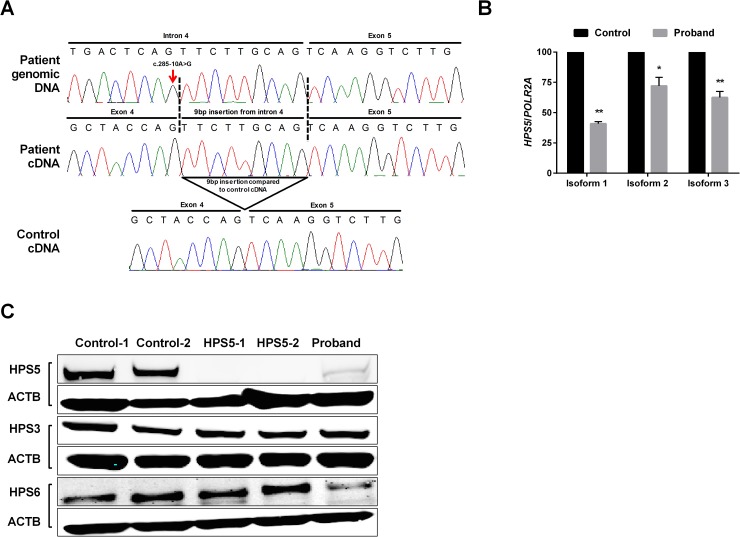
Molecular and expression analysis. (A) Chromatogram showing intronic mutation (c.285-10A>G) in genomic level (upper panel) and cDNA level (middle panel) of the patient. Lower panel shows normal cDNA sequence from control for comparison with patient. (B) Relative quantification of mRNA expression (three different isoforms) in patient derived dermal fibroblasts, compared to control. Error bar represents standard deviation from triplicates (*: p<0.05, **: p<0.01). (C) Western blotting results showing the expression of HPS5 in patients compared to control. Two different controls (Control-1 and Control-2), two additional patients with HPS5 (HPS5-1 and HPS5-2), and our proband were included. The level of protein expression was normalized with β-actin.

Genes associated with skin pigmentation were screened using targeted exome sequencing. Variants identified were filtered using the databases of dbSNP, ClinSeq and the Exac consortium (Broad Institute) with a minor allele frequency less than 0.05. Autosomal recessive model filter was employed due to the history of third degree consanguinity. Final analysis revealed a homozygous mutation, NM_181507.1; c.285-10A>G, in intron 4 of the *HPS5* gene; which was confirmed by Sanger validation (Fig 2A). This variant is reported in online Exac database (http://exac.broadinstitute.org/variant/11-18332490-T-C) as a heterozygous change with an allele frequency of 0.0001. No healthy homozygotes were reported for this variant. According to splice prediction softwares (NetGene2 server and Alamut), the variation is predicted to activate a cryptic acceptor splice site nine nucleotides before the usual acceptor site of intron 4. Parental DNA was unavailable. To confirm the prediction of altered splicing, we amplified patient cDNA derived from dermal fibroblasts using specific primers flanking exon 4 and exon 5. Sanger sequencing of the products revealed an insertion of nine nucleotides from the intronic region into the coding frame (Fig 2A), which is predicted to result in an in-frame insertion of three amino acids, serine, cysteine and serine (NP_852608.1; p.Ser95_Gln96insSerCysSer) in the highly conserved domain of HPS5.

### HPS5 expression in fibroblasts

*HPS5* has three main types of splice variants (Genbank), and the expression of each varies according to the type of tissue. At the transcript level, the mutation lies in the coding region of the longest isoform (variant 1; NM_181507.1), while the mutation resides in the 5’UTR of the two shorter isoforms (variant 2; NM_007216.3, variant 3; NM_ 181508.1). Since the position of the patient’s mutation varies among transcripts, we measured the expression of each isoform of *HPS5* using isoform-specific primers. Variants 1, 2, and 3 showed significantly decreased transcript levels to about 40%, 72% and 65% of normal, respectively (p<0.01, p<0.05, and p<0.01, respectively) (Fig 2B). Western blot analysis using a polyclonal antibody showed severely reduced HPS5 protein levels in the proband and two previously reported HPS-5 patients with loss-of-function mutations in *HPS5* [HPS5-1 (c.2593C>T; p.R865* and c.2624delT; p.L875Cfs*20) [12] and HPS5-2 (c.302_305delTTTG; p.V101Gfs*3 and c.1634+1G>A) [13]] compared to two separate controls (Fig 2C and S1 Fig). Evaluation of other BLOC-2 partners (HPS3 and HPS6) showed slightly reduced expression of HPS6 only in the proband (Fig 2C and S1 Fig). We also checked for the unaffected HPS complexes (HPS4 for BLOC-3, HPS7 for BLOC-1, and HPS2 for Adaptor Protein (AP)-3 complex) and found similar expression levels between HPS-5 patients and controls for all the proteins checked (S1 Fig).

### Expression and distribution of endo-lysosomal markers

**Fig 3:**
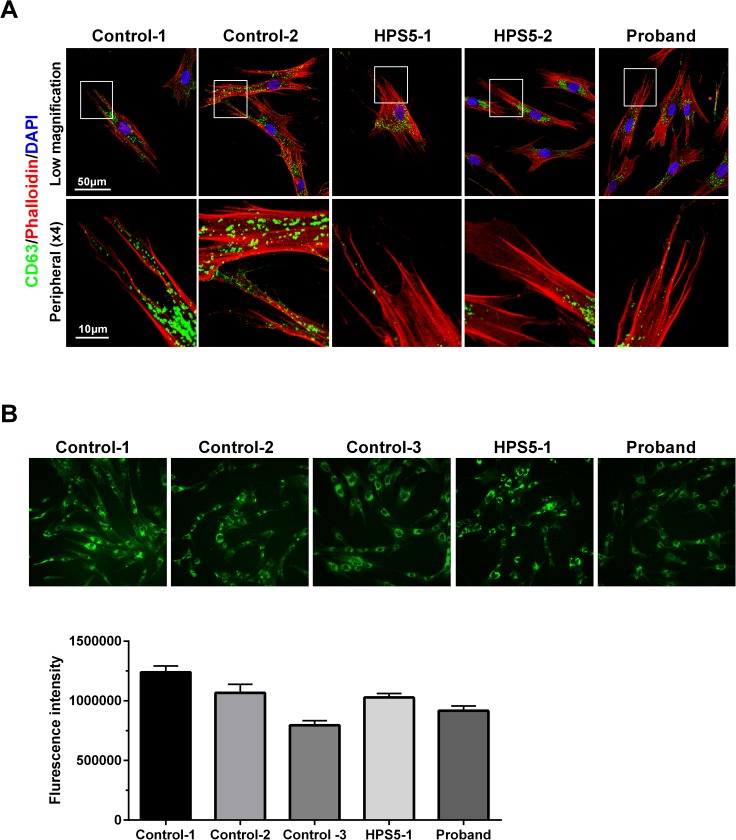
Cellular distribution of lysosomes. A. Distribution pattern CD-63 (green) in three HPS-5 patients including proband. Phalloidin (red) highlights cell boundary and DAPI (blue) stains nucleus. Upper panel shows lower magnification images showing perinuclear accumulation of CD63 in patients. Lower panel represents the higher magnification focusing on the peripheral region. B. Upper panel shows representative images for CD63 staining in 96-well format for quantification. Control image is a representative of three control cell lines used. CD63 is stained green. Lower panel is histogram representing the quantification of mean fluorescence intensity in samples.; ~6000 cells per well were seeded, and three replicates per cell line were measured. Error bars represent standard error of mean; no statistically significant differences were seen.

**Fig 4:**
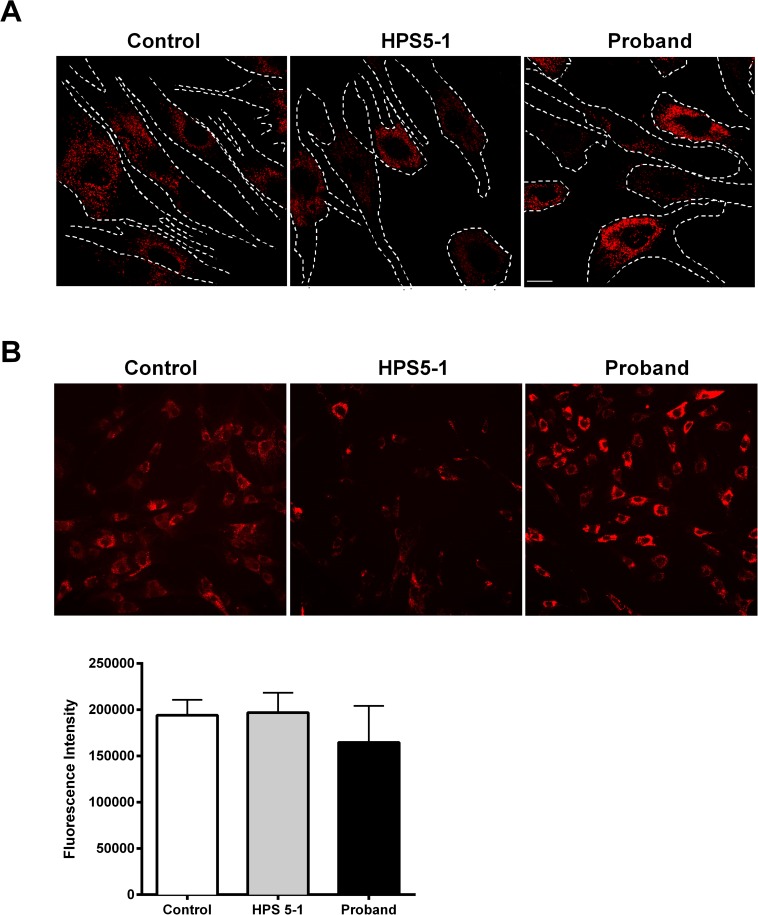
LysoTracker assay. LysoTracker red dye staining shows the distribution of acidic organelles including mature lysosomes of two HPS-5 patients, including the proband, compared to control. Cell boundaries are marked with white dotted line. Scale bar represents 200 px (pixels). Representative images for lysotracker staining in 96-well format for quantification. Control image is a representative of three control cell lines used. Lysotracker is stained red. A histogram representing the quantification of mean fluorescence intensity of samples is shown in the loer panel. ~6000 cells per well were seeded, and three replicates per cell line were measured. Data from control samples were pooled. Error bars represent standard error of mean; there were no statistically significant differences.

BLOC-2 participates in LRO biogenesis, such as melanosome and endosome maturation. Using patient-derived fibroblasts from three HPS-5 patients including the proband, we assessed the expression and distribution of early (EEA1), late (Rab7) and recycling (Rab11) endosomes and lysosomes (CD63). We found no difference in the distribution of EEA1 and Rab7 between control and patient cells (S2A Fig). Rab11 staining showed diffuse distribution through control and patient fibroblasts and suggested decreased fluorescent intensity in individuals with HPS-5 (S2A Fig). However, immunoblotting did not show a consistent reduction of Rab11 in HPS5 patients compared to two different controls (S2B and S2C Fig). The cellular distribution pattern of CD63 staining appeared to be altered in patients.: The CD63 signal is scattered throughout the cytoplasm in control and patient cell lines, while in patient cells this marker appeared less in the periphery of the cells and seemed to accumulate more in the perinuclear region when compared to controls (Fig 3A). Overall CD-63 intensity was not significantly different between patients and controls when cells were stained in a 96-well format for quantification (Fig 3B) Due to altered distribution of lysosome marker (CD63) by immunofluorescence, we next used LysoTracker dye staining to further assess the maturation and location of acidic organelles, including lysosomes, in individuals with HPS-5. We found these acidic organelles to be predominately around the nucleus in patient cells, in contrast to their more diffuse distribution in control cells (Fig 4A). However, the overall staining intensity was not significantly different between the two (Fig 4B).

## Discussion

HPS-5 is a rare subtype of HPS, with only eleven patients reported in the literature (Table 1). Our patient’s clinical manifestations of oculocutaneous albinism and bleeding are similar to those reported in other individuals with HPS-5. Our patient is an adult, and his evaluation showed no evidence of interstitial lung disease or pulmonary fibrosis. Notably, some of his alveolar macrophages appeared foamy. Although alveolar macrophages are foamy and activated in patients with HPS-1, this is the first report of bronchoalveolar lavage cells from a patient with HPS-5, thus contributing a novel view of the clinical management for HPS. As of now, HPS-5 patients, unlike patients with HPS-1, do not develop the fatal complication of pulmonary fibrosis. Although this patient did not have clinical evidence of lung disease, analysis of his bronchoalveolar lavage cells identified foamy alveolar macrophages, which resemble those observed in patients with HPS-1 and may indicate a response to a pro-fibrotic alveolar milieu. Our report suggests that clinicians should be vigilant in evaluating for possible lung disease in patients with HPS-5.

**Table 1:** *HPS5* mutations reported in the literature.

**Patient**	**Age in years**	**Sex**	**Ancestry**	**Allele 1**	**Allele 2**	**HPS5 mRNA expression in fibroblasts**	**HPS5 Protein expression in fibroblasts**	**Nystagmus onset**	**Eye color**	**Fundus**	**Ref**
**1**	3	M	Turkish	c.2026_2029del;p.Val676Metfs*8	c.2026_2029del;p.Val676Metfs*8	ND	NP	ND	ND	ND	[16]
**2**	10	M	1. English2. Irish 3. Dutch4. Swedish	c.2624del;p.Leu875Cysfs*20	c.2593C>T;p.Arg865*	decreased	NP	2 wks	Red	Pale	[12]
**3**	51	F	Swiss	c.1871T>G;p.Leu624Arg	c.3293C>T;p.Thr1098Ile	normal	NP	1 mo	Brown	Pale	[12]
**4**	43	F	Swiss (sister of Patient 3)	c.1871T>G;p.Leu624Arg	c.3293C>T;p.Thr1098Ile	normal	NP	ND	Brown	Pale	[12]
**5**	21	F	English and Irish	c.879dup;p.Lys294Glnfs*6	c.2928_2929dup;p.Thr977Argfs*15	decreased	NP	6 wks	Red	Pale	[12]
**6**	38	F	Turkish	c.888dup;p.His297Thrfs*3	c.888dup;p.His297Thrfs*3	NP	NP	ND	ND	ND	[17]
**7**	1	M	Mexican	c.1423del;p.Leu475Serfs*37	Not detected	NP	decreased	+	Brown	ND	[13]
**8**	3	M	Cuban-Venezuelan	c.302_305del;p.Val101Glyfs*3	c.1634+1G>A;p.Asp504Valfs*22	NP	decreased	+	Brown	ND	[13]
**9**	72	M	ND	c.1423del;p.Leu475Serfs*37	c.1423del;p.Leu475Serfs*37	NP	NP	Early in life	ND	Pale	[18]
**10**	30	M	Arabian	c.1618C>T;p.Gln540*	c.1618C>T;p.Gln540*	NP	NP	+	ND	Pale	[19]
**11**	20	F	Arabian	c.1618C>T;p.Gln540*	c.1618C>T;p.Gln540*	NP	NP	+	ND	Pale	[19]
**12**	27	M	Turkish	c.285-10A>G;p.Ser95_Gln96insSerCysSer	c.285-10A>G;p.Ser95_Gln96insSerCysSer	decreased expression of two shorter isoforms	decreased	+	Pale brown	Pale	This study

NP, Not performed; ND, not described

Except for two missense mutations (p.Leu624Arg and p.Thr1098Ile), all the pathogenic variants previously reported in *HPS5* were deleterious frameshift mutations (Table 1). In this study we report a novel in-frame insertion of three amino acids in the N-terminal region of HPS5. Unlike the reported cases, this mutation is found in an early coding region of the longest HPS5 isoform (NM_181507.1), and in the 5’UTR of the other shorter isoforms (NM_181508.1 and NM_007216.3). qPCR revealed decreased expression of all three isoforms correlating with our Western results (Fig 2B and 2C). BLOC-2, which is composed of HPS5, HPS3 and HPS6, has a role in LRO biogenesis in melanosome and endosome maturation [7] and likely plays a role in the transition between stage II and stage III of melanosome biogenesis, perhaps through its role targeting cargo delivery to the melanosome [14, 15]. Given the availability of dermal fibroblasts from this patient, we focused on the endo-lysosomal pathway in his cultured skin fibroblasts. HPS affects the biogenesis of lysosomes and lysosome-related organelles, while BLOC-2, specifically, has been shown to associate with early endosomal tubules. Thus, we wanted to investigate any aberrant dynamics in the endo-lysosomal system that may be present[14]. Our confocal microscopy results showed that the distribution of early (EEA1) and late (Rab7) endosomal markers is unaffected in the three HPS-5 patients (S2A Fig). Recycling endosome (Rab11) intensity was low in all three HPS5 patient tested, despite being normally distributed throughout the cell (S2A Fig). The lysosomal marker CD-63, was found to be localized predominantly around the nucleus (Fig 3) in patient cells, consistent with an earlier finding by Huizing et al [12]. This finding was further supported by the perinuclear distribution of acidic organelles, including mature lysosomes, that are stained with LysoTracker dye (Fig 4). Although CD63 staining seems to be more concentrated around the nucleus, a robust conclusion cannot be made by quantifying immunofluorescence alone. Future studies, such as organelle labeling, may help in this regard. In the past, Dennis et al. reported a similar nuclear accumulation of tyrosinase (TYR) and TYR-related protein 1, both melanosomal proteins, in BLOC-2-deficient mice and proposed a role for BLOC-2 in targeting endosomal tubules to melanosomes [15]. Taken together, our study suggests a role for BLOC-2 in endosome-mediated lysosomal maturation and processing in addition to its previously reported involvement in the melanosomal pathway.

In conclusion, we report a novel *HPS5* splice site mutation in an individual with HPS. Molecular and cellular findings prove pathogenicity of the mutation and confirm the role of BLOC-2 in endo-lysosomal dynamics.

## Supporting information

S1 Fig Immunoblotting for the BLOC-1,2,3 and AP-3 complexes in HPS5 patients and two different control dermal fibroblasts. (A) Western blotting results showing the expression level of HPS-5 and the other interacting partners of BLOC-2 (HPS3 and HPS6) along with a representative protein from each HPS complex (HPS2 for AP-3 complex, HPS4 for BLOC-3 complex and HPS7 for BLOC-1 complex) in patients compared to control. Two different controls (Control-1 and Control-2) and two additional HPS-5 patients (HPS5-1 and HPS5-2) were included along with our proband. Images include markers above and below the target band with estimated molecular weights for each protein. The level of protein expression was normalized with β-actin (ACTB).
(TIF)

S2 Fig Distribution of endosomal markers in control and patient cells. (A) Distribution pattern of organelle marker (EEA-1, upper panels; Rab 7, middle panels; and Rab11, lower panels) are shown in green Two control lines and three HPS-5 patients including proband were analyzed. Phalloidin (red) stains actin filament and highlights cell boundary and DAPI (blue) stains nucleus. Both lower magnification images and higher magnification images are shown. Of note, Rab11 shows less fluorescent intensity in the three patients compared to controls. (B) Quantification of Rab11 abundance by western blot in control and HPS-5 lines. Β-actin (ACTB) was used for normalizing total protein amount. Three replicates for western blotting were done. (C) Graph showing the quantification of bands detected by western. Error bars represent standard error of means.
(TIF)
