# Supplementary figures and images for: Cellular and molecular defects in a patient with Hermansky-Pudlak syndrome type 5
Source: PLoS One. 2017 Mar 15;12(3):e0173682. doi: 10.1371/journal.pone.0173682 (PMC5351877; doi:10.1371/journal.pone.0173682)

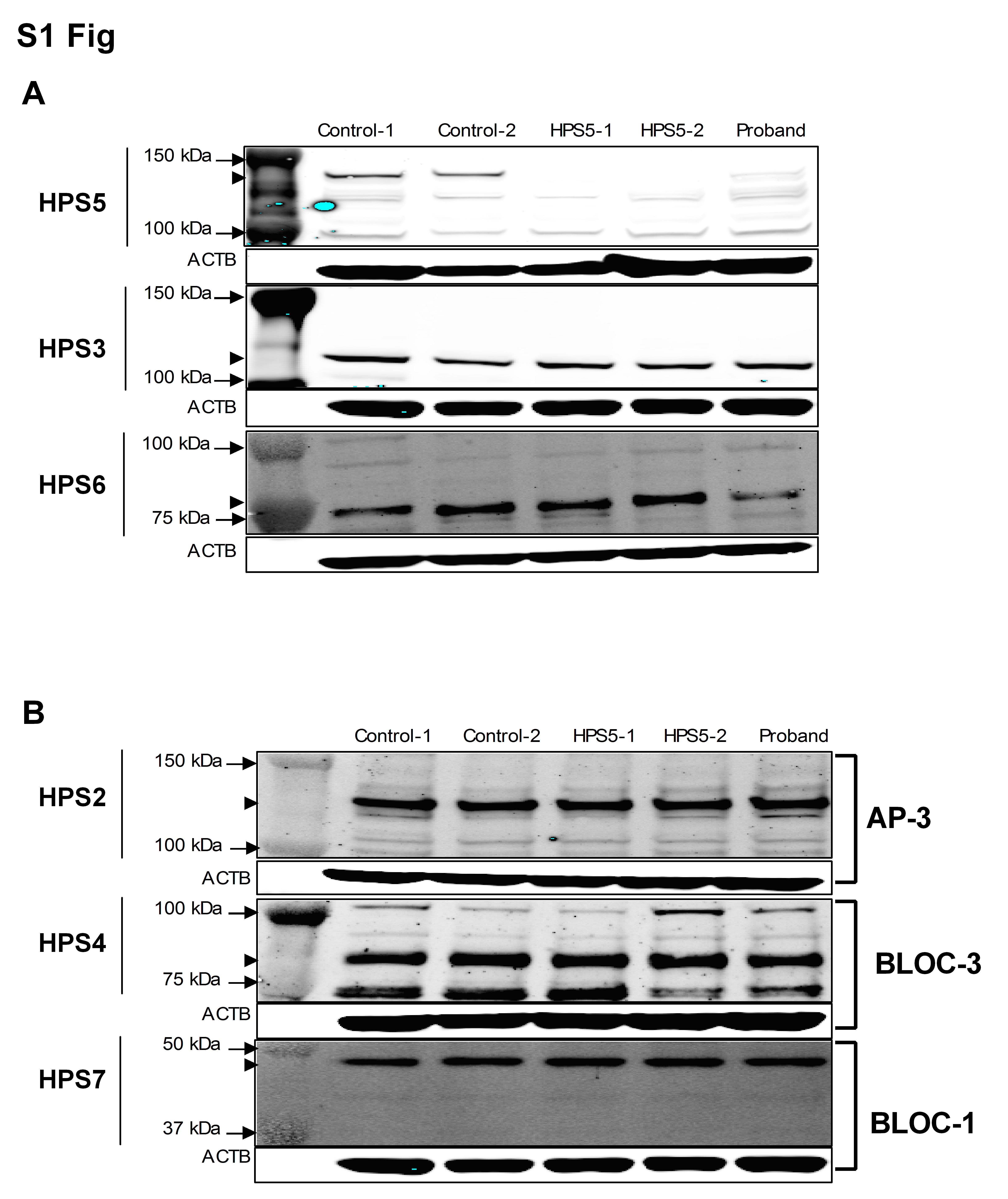

Supplement: S1 Fig — (A) Western blotting results showing the expression level of HPS-5 and the other interacting partners of BLOC-2 (HPS3 and HPS6) along with a representative protein from each HPS complex (HPS2 for AP-3 complex, HPS4 for BLOC-3 complex and HPS7 for BLOC-1 complex) in patients compared to control. Two different controls (Control-1 and Control-2) and two additional HPS-5 patients (HPS5-1 and HPS5-2) were included along with our proband. Images include markers above and below the target band with estimated molecular weights for each protein. The level of protein expression was normalized with β-actin (ACTB). (TIF) [file pone.0173682.s001.tif]

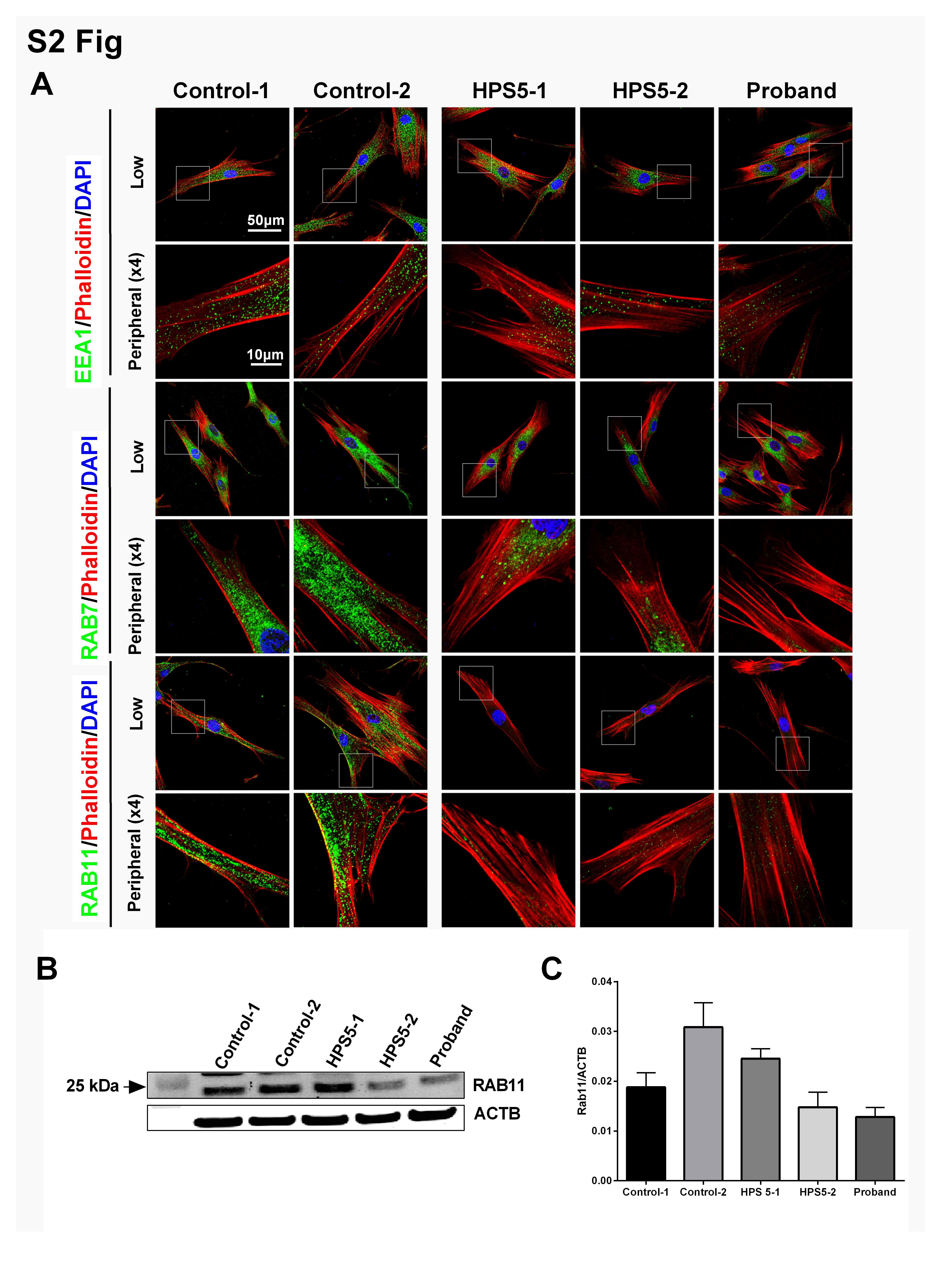

Supplement: S2 Fig — (A) Distribution pattern of organelle marker (EEA-1, upper panels; Rab 7, middle panels; and Rab11, lower panels) are shown in green Two control lines and three HPS-5 patients including proband were analyzed. Phalloidin (red) stains actin filament and highlights cell boundary and DAPI (blue) stains nucleus. Both lower magnification images and higher magnification images are shown. Of note, Rab11 shows less fluorescent intensity in the three patients compared to controls. (B) Quantification of Rab11 abundance by western blot in control and HPS-5 lines. Β-actin (ACTB) was used for normalizing total protein amount. Three replicates for western blotting were done. (C) Graph showing the quantification of bands detected by western. Error bars represent standard error of means. (TIF) [file pone.0173682.s002.tif]
